# Linking Climate Suitability, Spread Rates and Host-Impact When Estimating the Potential Costs of Invasive Pests

**DOI:** 10.1371/journal.pone.0054861

**Published:** 2013-02-06

**Authors:** Darren J. Kriticos, Agathe Leriche, David J. Palmer, David C. Cook, Eckehard G. Brockerhoff, Andréa E. A. Stephens, Michael S. Watt

**Affiliations:** 1 CSIRO Ecosystem Sciences, Canberra, Australia; 2 InSTePP, University of Minnesota, Department of Applied Economics, St. Paul, Minnesota, United States of America; 3 CRC National Plant Biosecurity, Bruce, Australia; 4 Scion (New Zealand Forest Research Institute), Rotorua, New Zealand; 5 Department of Agriculture and Food Western Australia, Bunbury, Australia; 6 The University of Western Australia, Crawley, Australia; 7 Scion (New Zealand Forest Research Institute), Christchurch, New Zealand; 8 HortResearch, Lincoln, New Zealand; University of Guelph, Canada

## Abstract

Biosecurity agencies need robust bioeconomic tools to help inform policy and allocate scarce management resources. They need to estimate the potential for each invasive alien species (IAS) to create negative impacts, so that relative and absolute comparisons can be made. Using pine processionary moth (*Thaumetopoea pityocampa sensu lato*) as an example, these needs were met by combining species niche modelling, dispersal modelling, host impact and economic modelling. Within its native range (the Mediterranean Basin and adjacent areas), *T. pityocampa* causes significant defoliation of pines and serious urticating injuries to humans. Such severe impacts overseas have fuelled concerns about its potential impacts, should it be introduced to New Zealand. A stochastic bioeconomic model was used to estimate the impact of PPM invasion in terms of pine production value lost due to a hypothetical invasion of New Zealand by *T. pityocampa*. The bioeconomic model combines a semi-mechanistic niche model to develop a climate-related damage function, a climate-related forest growth model, and a stochastic spread model to estimate the present value (PV) of an invasion. Simulated invasions indicate that *Thaumetopoea pityocampa* could reduce New Zealand’s merchantable and total pine stem volume production by 30%, reducing forest production by between NZ$1,550 M to NZ$2,560 M if left untreated. Where *T. pityocampa* is controlled using aerial application of an insecticide, projected losses in PV were reduced, but still significant (NZ$30 M to NZ$2,210 M). The PV estimates were more sensitive to the efficacy of the spray program than the potential rate of spread of the moth. Our novel bioeconomic method provides a refined means of estimating potential impacts of invasive alien species, taking into account climatic effects on asset values, the potential for pest impacts, and pest spread rates.

## Introduction

Invasive alien species (IAS) are a major problem worldwide. Despite the efforts of biosecurity agencies and an international phytosanitary legal framework for the management of invasion pathways, the rate at which these species are invading new territories appears to be increasing rapidly [1], [2]. Globalisation is having a major impact on the spread of crop pests with increased trade and travel providing new dispersal pathways for pests [3]. In addition to increasing the dispersal options for pests, globalisation of agriculture, silviculture, and horticulture is homogenising the distribution patterns of plant hosts across continents. In combination, these processes are increasing pest risk profiles in terms of both threats and vulnerabilities [4].

The rate of biological invasions has fuelled demands from biosecurity agencies for information on the potential distribution and impacts of IAS in terms of economics, human health and biodiversity. Climate has long been recognised as an important environmental determinant of the distribution of pests [5], [6], [7]. Process-based niche models, such as CLIMEX, have utilised these relationships to project the potential distribution and relative abundance of a wide range of invasive insects, weeds and pathogens under both current and future climatic conditions [8]–[13].

It is one thing for a biosecurity agency to know that a pest *could* establish within its jurisdiction, but another to be *able* and *willing* to respond to it should an incursion eventuate. IAS incursion responses are costly, and agencies have a finite capacity to respond. When faced with an incursion, they must decide whether to 1) attempt an eradication, 2) develop a campaign to slow the spread of the organism, 3) attempt to contain it, 4) allow the organism to spread without a coordinated response, and let land managers and local authorities manage the organism to reduce impacts to acceptable levels, or 5) allow the organism to spread, but mobilise a coordinated response such as a biological control programme [14], [15]. To assist them in this social resource allocation problem it is becoming more commonplace to see benefit cost analyses being used to guide these biosecurity response decisions [16], [17], [18].

Despite their common use in pest risk assessment, the potential of process-based niche models to inform these responses has not yet been fully realised. Although potential distribution is a useful input to pest risk assessment, in that it delineates an area in which the pest could potentially be problematical [19], [20], this information alone provides little insight into expected spatial and temporal variation in pest abundance, and consequent damage to crop species or other valuable assets. Such projections would be of considerable use to both biosecurity agencies and primary producers for quantifying and mitigating pest impacts and making decisions on allocation of resources. The development of bioeconomic models that can estimate impacts of pests on hosts, within a framework that spatially constrains pest distribution would therefore represent a major methodological advance.

It has been proposed recently that pine processionary moths are a species complex that consists of two species: *Thaumetopoea pityocampa* and *T. wilkinsoni* (Lepidoptera: Thaumetopoeidae) [21]. For consistency with the existing literature, we use here the previous nomenclature (*T. pityocampa*) to refer to the species complex, *T. pityocampa sensu lato*. *Thaumetopoea pityocampa* cause major defoliation of pines across southern Europe and around the Mediterranean Sea. Previous research has described the climatic requirements of *T. pityocampa* [22], [23] and this information was used to project the likely effects of a changing climate on its distribution in France [24]. Increases in outbreak severity and northward range expansion of the species, over the last few decades, are thought to be due to reduced winter mortality caused by a warming climate [23], [24].

Artificial woodlands appear more susceptible to attack by *T. pityocampa* than natural woodlands [25], and *Pinus radiata* appears more susceptible to attack by *T. pityocampa* than native European *Pinus* spp. [26], [27], [28]. Through time, defoliation appears to cycle [29] and this may be due to an unknown factor that reduces larval survival on plants that have suffered repeated defoliation [30].

Although the relationship between climate and host damage has not yet been quantified, defoliation of pines by *T. pityocampa* has been shown to result in growth reductions of up to 83% [29]. Should *T. pityocampa* be accidentally introduced into the southern hemisphere where its preferred host *P. radiata* is the most widely planted of all plantation species [31], there is considerable scope for serious economic damage. New Zealand is possibly highly vulnerable to damage from *T. pityocampa* as it has a significant plantation resource (1.8 million ha), of which approximately 90% comprises *P. radiata* [31].

In this contribution we demonstrate a novel analytical framework combining ecological and economic modelling, to estimate the economic impacts of an invasive organism as a basis for informing policy-level decision-making. We use New Zealand as a case study, and simulate the impact of an invasion by *T. pityocampa* on volume and present value of the plantation estate under current climate, assuming control or no control of *T. pityocampa* using insecticide. Finally, we discuss the utility of this case study as a generic approach for quantifying economic impacts of pests on important crop species.

## Materials and Methods

The analytical framework consists of five components, 1) a climate based host productivity model, 2) a niche model describing the climate suitability for the pest species, *T. pityocampa*, 3) a climate-based host damage function, 4) a spread model and 5) a partial budget economic model.

### Climate-based Host Growth Model

**Figure 1:**
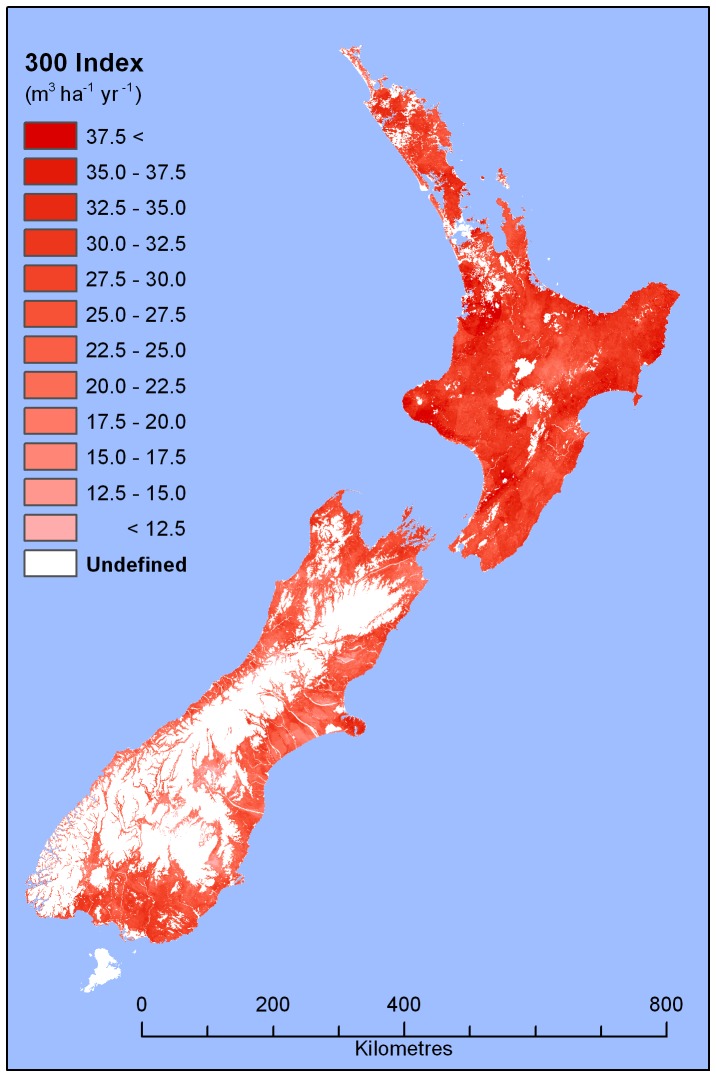
Spatial variation in 300 Index (mean annual volume increment at age 30 for a standard regime grown at 300 stems ha^−1^) for *Pinus radiata* in New Zealand [32]. Projection: New Zealand map grid.

The 300 Index defines the volume mean annual increment (MAI) for *P. radiata* at age 30 years with a reference stem density of 300 stems ha^−1^ (Fig. 1) [32], [33]. We used the 300 Index to define site productivity, as this provides a standardised measure of volume that is independent of age and stand density [33]. For the 300 Index, growth is expressed on an annual basis (averaged over a 30 year period), which corresponds well to the frequency over which damage is typically recorded. Another advantage of using the 300 Index is that volume translates readily into fiscal value, allowing potential economic losses resulting from *T. pityocampa* damage to be estimated.

The MAI described by the 300 Index is a total annual stem volume flux. There is typically 15% breakage in harvesting, and approximately 10% of stands are unstocked (MW, pers. obs.). Therefore estimates of MAI for stands both with and without *T. pityocampa* were reduced by 25% per hectare to determine the merchantable MAI. All economic analyses of damage described below relate to the merchantable MAI.

### Niche Modelling for Thaumetopoea Pityocampa

#### The CLIMEX model

CLIMEX is a dynamic species niche model that integrates modelled weekly responses of a population to climate in order to create a series of climatic suitability indices that can be mapped or graphed [34], [35]. CLIMEX uses an annual Growth Index (GI_A_) to describe the potential for population growth as a function of soil moisture and temperature during favourable conditions. It uses up to eight stress indices (cold, wet, hot, dry, cold-wet, cold-dry, hot-wet and hot-dry) to simulate the ability of the population to survive unfavourable conditions. CLIMEX also includes a mechanism for defining the minimum amount of thermal accumulation (number of degree days) during the growing season that is necessary for population persistence (PDD).

The growth and stress indices are calculated weekly and then combined into an overall annual index of climatic suitability, the Ecoclimatic Index (EI), which gives an overall measure of the potential of a given location to support a permanent population of the species [35]. The EI ranges from 0 for locations at which the species is not able to persist, to a theoretical maximum of 100 for locations that are climatically perfect for the species [35]. Due to seasonality in climate, it is usually only near the equator where climatic conditions are stable enough that values of 100 are ever attained [12].

The stress parameters for CLIMEX models are generally fitted to known distribution data using an iterative manual process. This involves adjusting growth and stress parameters and then comparing model results to the known distribution of the species, and including consideration of any additional information about the species being modelled, such as minimum and maximum temperatures for growth. In setting these parameters, consideration is also given to the biological plausibility of the selected parameters. This process allows models to be developed that accord with the known biology of the species. The growth parameters of CLIMEX models generally have less influence on the modelled potential range of the species (EI ≥1) than they do on the climate suitability patterns *within* that range. Although growth parameters can be fitted from simple presence records, direct observation of the species growth response to climatic variables are most useful for estimating these values. Another option is to use observed phenological patterns to inform these growth responses [36].

#### Fitting CLIMEX parameters for *Thaumetopoea pityocampa*

Due to its pest status in Europe, there has been a significant amount of research effort applied to understanding the current and potential distribution of *T. pityocampa* in its native range, as well as the factors limiting it, including an *ad hoc* correlative model [22], [24] and laboratory studies into survival and feeding behaviour at low temperatures [37], [38]. Whilst the latter studies may inform niche modelling for international pest risk assessment, the ability of a correlative species distribution model to provide robust projections into a novel climate is highly questionable [39], [40].

In fitting the CLIMEX parameters for the *T. pityocampa* model, three sources of data were considered: distribution data that indicated locations where the climate was suitable for persistence, the published results of a set of laboratory experiments, and previous modelling of its *Pinus* spp. hosts. The model accounted for the effects of the nest building behaviour of *T. pityocampa* on its temperature relations, which allows the larvae to overwinter without entering diapause or quiescence [38]. The larvae build silk nests on sun-exposed branches in which they rest during the day. If night-time temperatures are suitable they forage at night. This behaviour and biogenic heating of the nests means that the larvae experience a microclimate that is significantly different from the Stevenson screen air-temperatures used to develop long-term climatic records [41]. One effect of this factor is that fitted relationships between presence and air temperature could result in an accurate model, but not be indicative of the true temperature-presence relationship.

The climate data used in the CLIMEX modelling was the CliMond 10′ global historical dataset centred on 1975 (CM10_1975H_V1_WO) [42]. This dataset includes monthly average data for minimum and maximum air temperature, precipitation, and relative humidity recorded at 09∶00 and 15∶00. Whilst the climate averages in the CM10_1975H_V1_WO dataset are centred on 1975, they include data from 1951–2000 for precipitation [43], and 1961–1990 for temperature and humidity [44].

**Figure 2:**
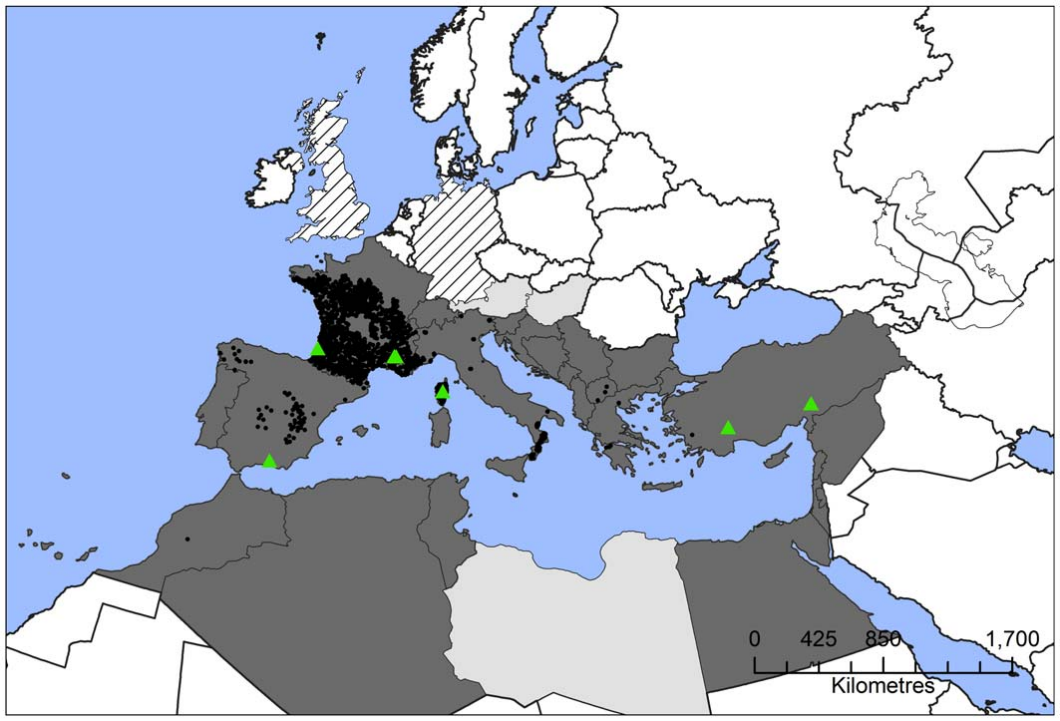
Known distribution of *Thaumetopoea pityocampa* in Europe, Asia and North Africa, combining records from before and after 1990. Black circles indicate known locations for *T. pityocampa,* green triangles are locations from which impact data has been reported. Countries are shaded to indicate areas where *T. pityocampa* has been reported as occurring regularly (dark grey), seldom found (light grey), presence unknown (cross-hatched) and absent (white). Projection: Albers equal-area.

Distribution data for *T. pityocampa* (Fig. 2) were assembled from a literature review and various databases including the entry for processionaria del pino in the *Atlas Linguistico Y Etnographico de Castilla-La Mancha* [21], [25], [45], [46], [47]. Noting the recent range expansion by *T. pityocampa*, presumably in response to climate warming [23], [24], we took care to define a geographic dataset describing species presence that corresponded as closely as possible to the climate dataset used for fitting our niche model (Fig. 2). The CLIMEX stress parameters were fitted to this pre-1990 European data and all of the Middle-East and North African data. The parameters used to model the distribution of *T. pityocampa* are described in Table 1. The fitted parameters were applied to the same historical climatology dataset for New Zealand to define its potential distribution there. This method ignores both the recent range expansion of *T. pityocampa* and the recent climatic changes [23]. Therefore, the resulting model should be adequate and appropriate when it is applied to different climatic datasets. By projecting the potential range in New Zealand using the historical climate, we are likely to be biasing our results slightly conservatively.

**Table 1:** CLIMEX parameter values used for *Thaumetopoea pityocampa*. Parameter mnemonics are taken from Sutherst *et al.* (2007b).

Index	Parameter	Value^a^
Temperature	DV0 = lower threshold	5.6°C
	DV1 = lower optimum temperature	18.0°C
	DV2 = upper optimum temperature	25.0°C
	DV3 = upper threshold	30.0°C
Moisture	SM0 = lower soil moisture threshold	0.25
	SM1 = lower optimum soil moisture	0.40
	SM2 = upper optimum soil moisture	0.80
	SM3 = upper soil moisture threshold	2.00
Cold stress	TTCS = temperature threshold	−4°C
	THCS = stress accumulation rate	−0.05 Week^−1^
	DTCS = degree day threshold	17.0°C Days
	DHCS = stress accumulation rate	−0.00025 Week^−1^
Heat stress	TTHS = temperature threshold	32.0°C
	THHS = stress accumulation rate	0.005 Week^−1^
Dry stress	SMDS = threshold soil moisture	0.2
	HDS = stress accumulation rate	−0.005 Week^−1^
Generations	PDD = degree-day threshold^b^	600°C Days

a Values without units are dimensionless indices of a 100 mm single bucket soil moisture profile.

b Minimum annual total number of degree-days above DV0 needed for population persistence.

#### Growth parameters

The temperature growth parameters for *T. pityocampa* were mostly derived from published observations and development rate experiments. The lower temperature limit for development DV0 was set to 5.6°C in order to allow sufficient development of the moth at apparently suitable northern European locations (Fig 2). This is consistent with the rule developed by Battisti *et al.* [23], whereby for feeding to occur, daytime temperatures need to reach 9°C (the activation temperature) followed by a night time temperatures that do not fall below 0°C. The lower and upper temperature optima (DV1 and DV2) were set to 18 and 25°C respectively, representing a bracketing of the upper value for the maximum temperature under which processions occur [48]. The upper temperature limit for development (DV3) was set to 30°C, as at elevated temperatures around 30 to 32°C no larval activity is noted [41] and in some cases mortality is observed [48].

The lower soil moisture for growth (SM0) was set to a value of 0.25 that allowed appropriate persistence at the dry range limits in North Africa. The upper soil moisture level for growth (SM3) was taken from a previous model indicating the maximum soil moisture suitable for *Pinus* spp. [49]. The soil moisture optima were fitted to give a growth index that broadly agreed with observations of maximum tree growth impact throughout the suitable range.

#### Stress parameters

Geographical distribution data most strongly influences the fitting of the stress parameters, as these most directly influence the ability of a species to persist in an area. The primary limiting factor for *T. pityocampa* in Europe is cold stress. Two forms of cold stress appear to affect *T. pityocampa* in different parts of its range. Whilst the supercooling point of individuals of *T. pityocampa* has been shown to be −7°C [38], the ecological limit for colony survival are temperatures of approximately −5°C [37]. A cold stress temperature threshold of −4°C was found to accord well with the distribution records. Since larvae need to forage during the cool season, but may only do so when their nests become warm, a degree day cold stress was fitted to the northern range boundary in Europe. This mechanism required 15 degree days per week above 5.6°C to stave off cold stress. This set of degree day parameters is likely to be consistent with the observation by Hoch *et al.* [38] that feeding did not occur until daily temperatures reached 9°C day/0°C night. The difference in thresholds may be because Hoch *et al.* (2009) used a square wave day/night time temperature pattern, while a circadian daily temperature cycle occurs in the field and is used within CLIMEX. Another possible reason for this slight disparity is differences between instantaneous nest temperatures and long-term average screen temperatures as they relate to survival and feeding behaviours of *T. pityocampa*.

According to the model, the combination of lethal cold temperature and degree day cold stresses limited the ability of *T. pityocampa* to occupy sites in the Pyrenees and north of the Massif Central in southern France. It also prevented occupation of southern Britain. Seasonal dry stress appears to affect *T. pityocampa* in central Spain and the south of Italy and Greece. Dry stress becomes limiting in northern Africa where the dry stress parameters were fitted to the single known point location at Marrakech in Morocco [47]. Heat stress appears to affect *T. pityocampa* in parts of southern Spain and at Marrakech. The heat stress parameters were fitted to limit its distribution in these regions. The paucity of data in these hot dry regions means that model results in the hot and dry extremes should be considered indicative, rather than reliable.

**Figure 3:**
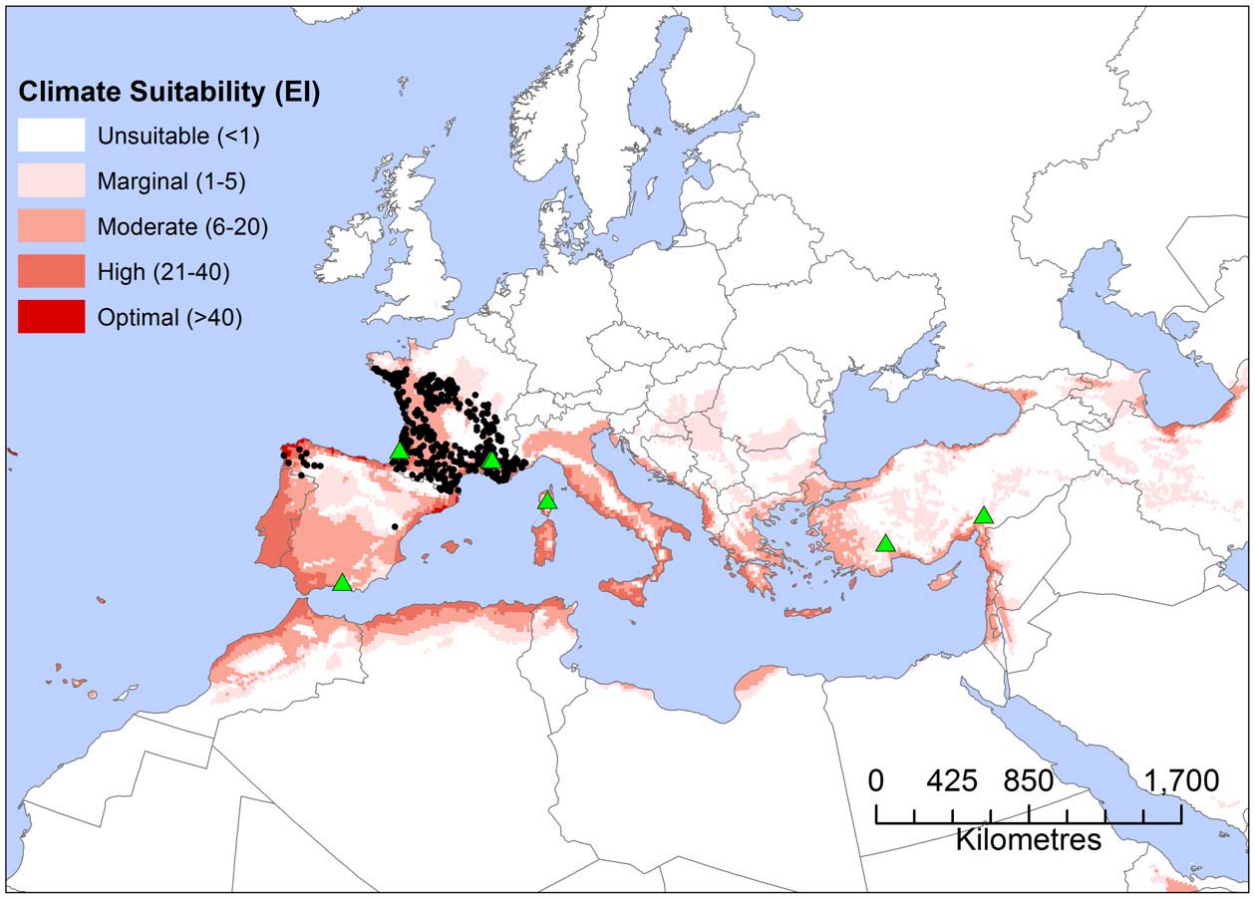
Europe showing known and potential range of occurrences of *Thaumetopoea pityocampa.* *Thaumetopoea pityocampa* up to 1990 (black dots) and modelled climatic suitability for *T. pityocampa* under historical (1961–1990) climate averages as modelled using CLIMEX Ecoclimatic index (EI). Projection: Albers equal-area. Green triangles are locations from which impact data has been reported.

Using the parameter values described in Table 1, the projected potential distribution within Europe, Asia and north Africa (locations with an EI≥1) fitted known observations reasonably well (Fig. 3). The shape of the northern range limit is well characterised, and was clearly reproduced in the CLIMEX model. The few reported location points for *T. pityocampa* that did not accord with the model results were mainly found in areas of dissected terrain (e.g. the northern limits of the Massif Centrale in France). This same model misfit problem was encountered by Robinet *et al.*, [24] using a generalised additive model, suggesting that there is a problem with the distribution data, the climate data or both around the massif Central and the pre-1990 northern French range boundary for *T. pityocampa*. Another option is that these points could fall within the modelled climatically suitable zone if the model were applied to a sufficiently fine resolution climatological surface. This scaling issue has been explored previously [50], and it is unlikely to have a serious impact on the resulting model as it affects marginally suitable locations covering a small area in dissected terrain. Whilst the hot and dry range limits of *T. pityocampa* are less well defined with occurrence records, the model nonetheless matches the known pre-1990 distribution throughout the Mediterranean Basin.

### Climate-based Growth Impact Function

Values for variables describing the annual percentage of growth decrease (radial, diametric and circumference) over the five years following attacks by *T. pityocampa* compared to non-attacked trees were used in this study to define growth losses attributable to *T. pityocampa*. Reports from fourteen sites across Europe were used to build the relationship between modelled EI values and the level of growth impact on a range of pine species [29], [51]–[54]. These pine species did not include *P. radiata*, which is a preferred host and highly susceptible to attack and, because of this, rarely planted in Europe today, except in Spain.

**Figure 4:**
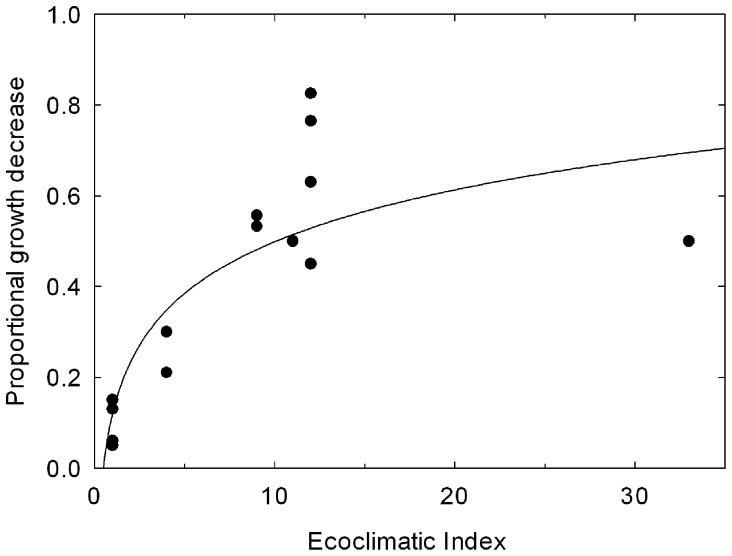
Growth impacts from *Thaumetopoea pityocampa* as a function of climate suitability modelled using CLIMEX. The location of sites with reported growth impacts used to develop the relationship are indicated in Figure 2.

Values of EI at these locations were extracted and regressed against the reported damage values. Where necessary the reported damage values were transformed into a proportional volume growth reduction. For the fourteen sites, EI accounted for 67.9% of the variance in the damage (Fig. 4). The following fitted logarithmic function was used to model annual proportional growth losses (*D_i_*) as a function of climate suitability for *T. pityocampa* (EI) in each 10′ cell (*i*):
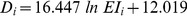
(1)


The curvature of the fitted function is somewhat sensitive to the point with the highest climate suitability (50% growth impact with an EI of 33). However, including this point in the analysis ensures that the function does not reach 100% impact (a biologically reasonable feature).

As *P. radiata* is more susceptible to *T. pityocampa* than many other pine species [26], [27], [28], we assumed that the damage function described above would provide a conservative estimate of damage. The damage function (Equation 1) was applied to the Ecoclimatic Index results for each 10′ climate station cell in New Zealand to create a polygon fishnet dataset of *P.radiata* potential proportional growth reduction.

### Spread Modelling

#### Model overview

Given the lack of knowledge about the likely point of entry of *T. pityocampa* in New Zealand, we make no assumptions about where the initial incursion occurs. Similarly, because of the large inherent uncertainty regarding the spread of invasive organisms over long time scales [55], [56] we make no attempt to explicitly simulate the spread of *T. pityocampa.* Instead, we use the spatially implicit spread model developed by Waage *et al.* [57]. In this model, the spread function is applied to the total area of host plantations, and subsequently, the total proportion of this area that is occupied at time *t* is used to apportion the cell-specific growth impact function to all cells containing pine plantations based upon their areal extent.

The spread model of Waage *et al.* [57] assumes that an invasive pest species spreads by a diffusive process such that area occupied by the population expands following the function:
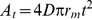
(2)


Where 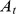 is the area occupied at time 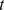; 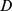 is the population diffusion coefficient (e.g. km^2^ yr^−1^); 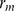 is the intrinsic rate of population growth (yr^−1^) specified as a PERT function [58], [59], [60]. This spread model assumes that the population is in a homogenous environment and expands at an equal rate in each direction.

As the area involved in an initial site expansion and the population density within that area increases, so too does the likelihood of a random satellite outbreak some distance from the original site:
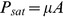
(3)


Here 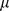 is the rate of satellite generation specified as a PERT function, and 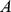 is the occupied area. Once generated, each satellite population grows and expands in the same manner as the original population. The total area of the original site occupied and the number of satellites grows until 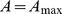 (maximum habitable area), at which point total area occupied remains constant.

#### Simulations

In total, five scenarios were examined. The first two assumed that *T. pityocampa* was fully dispersed throughout suitable areas in New Zealand, and as such make no assumptions around spread. These two equilibrium scenarios are *Equilibrium No Control* (no control) and *Equilibrium with Control* (infestations are controlled using *Bacillus thuringiensis* var. Kurstaki, Btk). In addition three invasion spread scenarios were simulated: 1) No control: an unmanaged invasion by *T. pityocampa*, 2) Little control: invasion by *T. pityocampa* that is managed ineffectively [maximum rate of population growth (*r_m_*) reduced by 50%], and 3) Good control: invasion by *T. pityocampa* that is managed effectively (*r_m_* reduced by 95%). For the three scenarios in which Btk is applied it is assumed that the insecticide completely negates the negative impact of *T. pityocampa* on growth over the area occupied by the moth.

For each invasion scenario a set of simulations were run using the spread model of Waage *et al.* [57]. Parameters within the spread model were specified as distributions, and a Latin hypercube sampling algorithm used to sample from each distribution. In each of 2 000 model iterations one value was sampled from each distribution within the model and calculations performed using this set of parameters. To test their sensitivity, parameters *r_m_* (the maximum rate of population growth) and *μ* (the rate at which satellite populations are generated each year) were then sampled from uniform distributions described in Table 2 with a maximum (minimum) of +95 per cent (−95 per cent) of their mean values using Monte Carlo simulation.

**Table 2:** Parameter values used in spread simulations using the spread model of Waage *et al.* [57].

Scenario	Parameter		
	*r_m_* (year^−1^)	*μ* (year^−1^)	*D* (ha year^−1^)
No control	0.2–0.5	0–0.00001	1 996
Little control	0.1–0.25	0–0.00001	1 996
Good control	0.01–0.025	0–0.00001	1 996

*r_m_* is the maximum rate of population growth *μ* is the rate at which satellite populations are generated each year, and *D* is the diffusion coefficient.

### Specification of Parameter Values in Models

#### Spread rate

Whilst it is possible for male moths of *T. pityocampa* to fly 20 km [61], female moths are short-lived, and can only fly short distances before ovipositing a single batch of eggs [62], [63], [64]. An analysis of the historical spread of *T. pityocampa* over 32 years in the comparatively flat Paris Basin indicated an average annual radial rate of spread of 2.7 km yr^−1^, though more recently rates of 5.6 km year^−1^ have been observed [23]. When translocated outside of the current range, *T. pityocampa* larvae were able to survive conditions colder than presently occupied, which indicates that the observed spread could be limited by the dispersal rates of the female moths, and the present range is lagged behind the retreating climatic limits. The diffusion coefficient in Equation 2 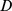 can be derived from the Mean Dispersal Distance (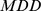) [65]:
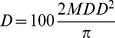
(4)where *D* is the diffusion coefficient (ha yr^−1^), and *MDD* is the average radial rate of spread (5.6 km year^−1^), which yields a value of 1 996 ha year^−1^.

In a survey of spread rates for 27 Lepidoptera, Waage *et al.* [57] noted that only one, *Pieris rapae* exhibited significant acceleration in its range expansion that indicated that long-distance jump dispersal was likely to be a significant factor in its invasion. In that survey, observed rates of radial range expansion were predominantly less than 10 km yr^−1^, which agrees with the estimate from Battisti et al [23].

#### Fecundity and population growth

Pimentel *et al.* [64] undertook a study of the population dynamics of *T. pityocampa*. They found that the fecundity of a winter population of *T. pityocampa* was 197.4±45.4, with 98.4±1% viability. Whilst Pimentel *et al.* [64] noted approximately 13% parasitism, this was in the native range, and is likely to overestimate the attack rates in a new environment unless the parasitoids are also translocated with *T. pityocampa*. Predation resulted in approximately 5% of nests having no survivors, with survival of larvae in other nests of 32.2±22.8%. Taken together, these results indicate an average value for the net reproductive rate, *r* of approximately 1.47 year^−1^, derived using Equation 5.
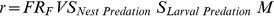
(5)where *F* is average fecundity of each egg batch (197) [64], *R_F_* is the (assumed) female sex ratio for eggs (0.5), *V* is average egg viability (0.98) [64], *S_NestPredation_* is the assumed average fraction of nests with at least one survivor (0.95), *S_LarvalPredation_* is the average fraction of larvae surviving predation (0.32) [64], and *M* is the assumed mating success rate (0.05). The value of *M* for the No Control scenario was selected after consideration of the observed mating success rate for gypsy moth (*Lymantria dispar* L.) under low density conditions such as those experienced in the region of the invasion front [66]. The biotype of *L. dispar* studied by Robinet *et al.* shares with *T, pityocampa* the qualities of highly mobile males and sessile females [66]. In *T. pityocampa*, the females lay a single batch of eggs; colonies are formed from one or several batches of eggs [64]. Note that the reproductive rate variable *r_m_* included in the model of Waage *et al.* [57] is equal to *r* –1 as it is included within a difference equation framework, hence a value of 1.45 for *r* is equivalent to a value of 0.45 for *r_m_*. The carrying capacity of pine forests for *T. pityocampa* is estimated to be 0.95 nests per tree×1 000 trees ha^−1^×1 adult female per nest. It was assumed that only one female reaches the adult stage.

The annual rate of generation of satellite populations of *T. pityocampa* (*μ*) was very low (Table 2). This reflects the assumption that during an incursion, plantation hygiene would minimise, but not eliminate, long distance dispersal of eggs or pupae well ahead of the invasion front, creating new invasion foci.

### Partial Budget Model of Economic Damage from Thaumetopoea pityocampa

The 300 Index spatial dataset describing *P. radiata* productivity throughout New Zealand [32] and a shapefile defining the spatial extent of the New Zealand pine plantation estate were spatially intersected with the 10′ cells from the CLIMEX analysis. These paired values were extracted into an Excel spreadsheet for subsequent analyses.

The total potential loss to pine plantations from *T. pityocampa* in m^3^ yr^−1^ was calculated using the following equation:
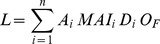
(6)(where *A* is the area (m^2^) planted to forest in each cell and *MAI_i_* is the area-weighted average 300 Index value in m^3^ ha^−1^ yr^−1^_,_ and *O_F_* is the outbreak or attack frequency reported by Laurent-Hervouet [29] for *T. pityocampa* (0.5 yr^−1^), and *n* is the total number of 10′ cells in New Zealand that are climatically suitable for *T. pityocampa* under historical conditions.

The present gross value of this production loss *GVP_L_*, was calculated as,
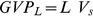
(7)where *V_S_* is the stumpage value (presently NZ$55.00).

We assumed that Btk was applied aerially to infested forests throughout the 30 year period, at a cost of NZ$120 ha^−1^ (ca. 60 € ha^−1^) [67]. It was also assumed that Btk completely negates any detrimental impact of *T. pityocampa* on crop volume growth.

Under each scenario, the impact of *T. pityocampa* on the tree growth rates across the New Zealand plantation estate was then estimated using discounted cashflow analysis. Analyses were undertaken to estimate discounted annual losses over a 30 year period from 2010 to 2040 (the invasion was assumed to start in 2010). In this analysis, it was assumed that forest rotations and harvests would occur on a rolling basis and so the forest age class would have a stable distribution. The discount rate used was 7% *per annum*.

## Results

### Potential Distribution of Thaumetopoea pityocampa within Current Plantations

**Figure 5:**
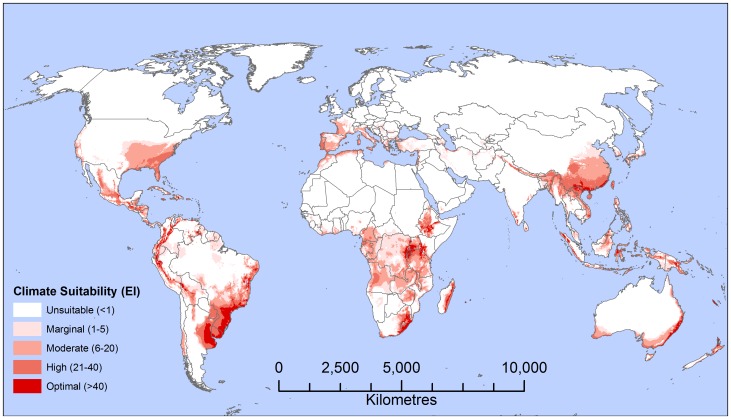
Global climate suitability for *Thaumetopoea pityocampa* modelled using CLIMEX Ecoclimatic index (EI). Projection: Robinson.

To verify the climate suitability model for *T. pityocampa*, the CLIMEX Ecoclimatic Index was compared with the reported distribution in Europe and North Africa (Fig. 3). The visual fit is satisfactory, with good model specificity (few false positives, Fig. 3). Elsewhere in the world, the CLIMEX model indicates that the potential range includes warm temperate, Mediterranean and moist sub-tropical climates. Notably, the most favourable climates for *T. pityocampa* appear to lie outside of its native range, in eastern South America, pockets of southern and central eastern Africa, southern China and eastern Australia (Fig. 5).

**Figure 6:**
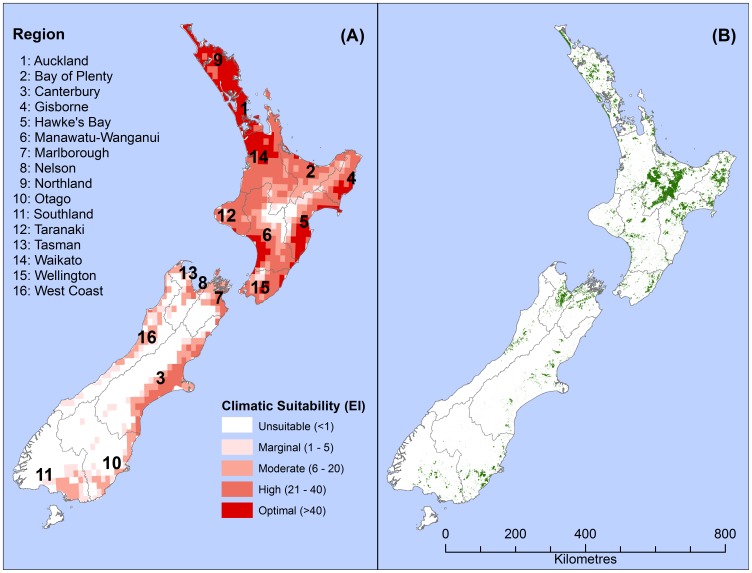
New Zealand map showing: (a) climatic suitability, by region, under current climate for *Thaumetopoea pityocampa* using CLIMEX Ecoclimatic Index (EI), and (b) existing (green) pine plantations [69]. Projection: New Zealand map grid.

In New Zealand, under current climate, most of the coastal North Island and a strip along the east coast of the South Island appear suitable for *T. pityocampa* (Fig. 6A). With the exception of part of the central North Island forests, most plantations in the North Island were projected to have a suitable climate for the moth (Fig. 6B). In total 65% of the total plantation area within New Zealand was projected to be suitable for *T. pityocampa*. The proportion of plantation area suitable for *T. pityocampa* declined with latitude (Fig. 6A).

### Rate of spread of Thaumetopoea pityocampa

**Figure 7:**
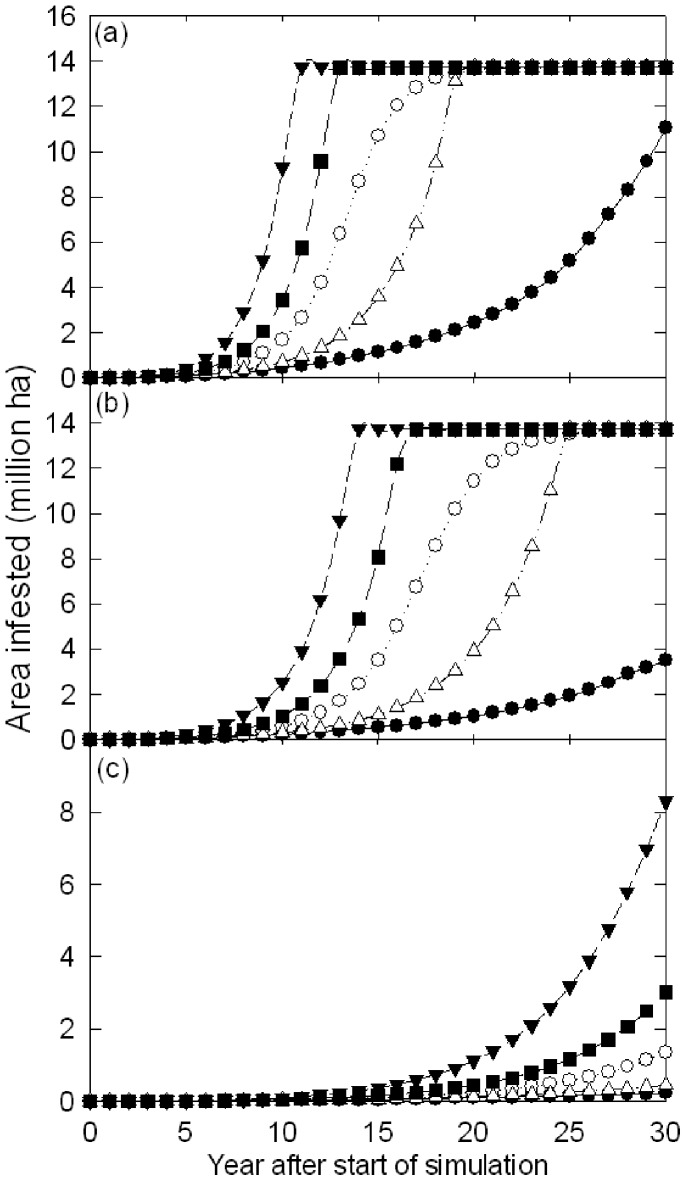
Change in area infested by *Thaumetopoea pityocampa* from the simulation start under the (a) no control (b) little control and (c) good control spread scenarios. On all figures the lines represent trajectories from spread simulations showing the minimum, (filled circles), maximum (filled triangles), 5^th^ (open triangles), 50^th^ (open circles), and 95^th^ (closed squares) percentiles. Note the variation in scale between figure panels.

The modelled rate of spread of *T. pityocampa* varied widely between scenarios (Fig 7A). Examination of the median simulations showed that 20 years after the simulation starts the moth occupies, 99% (Fig. 7A), 83% (Fig. 7B) and <2% (Fig. 7C) of the climatically suitable area, respectively, for the no control, little control and good control scenarios respectively. Although there is variation around the average for these scenarios, this variation is much smaller than the differences that exist between scenarios (Fig. 7). Under the least conservative no control scenario, sampled from the 95 percentile, the moth completely occupies the climatically suitable area within 13 years of the start of the simulation (Fig. 7A).

Variation in production losses with and without *Thaumetopoea pityocampa.*

### Assuming Full Occupancy of the Climatically Suitable Area

The Equilibrium No Control simulation indicates that if the moth was presently fully dispersed throughout its potential range and left untreated, merchantable timber production would be reduced nationally by 29.9% or 8.8×10^6^ m^3^ yr^−1^, which is equivalent to NZ$482 million yr^−1^. In the absence of control efforts, potential regional losses are likely to vary from 76% of harvest revenue in Northland forests to nil in some Southland forests. This figure represents the annual recurrent losses in 2010 terms that we could expect in the absence of any efforts to reduce the damage.

Under the Equilibrium with Control scenario, expected annual financial losses are NZ$72 million yr^−1^. Given that volume losses are assumed to be nil in stands treated with Btk this figure represents the annual cost of spraying the affected area with Btk. Comparing the two scenarios indicates an average net saving of 85% through the application of control measures.

Under the spread scenarios, without any controls, the total present value (PV) losses ranged from NZ$1,550 M to NZ$2,560 M (Table 3). Under the assumption that *T. pityocampa* is controlled using aerial application of Btk, the combined costs due to production losses and spray costs were reduced significantly, but nonetheless remain substantial, particularly if the control is only moderately effective (Table 3).

**Table 3:** Present value of damage to commercial forestry operations in New Zealand due to the spread of *Thaumetopoea pityocampa* over 30 years.

Spread Scenario	Present value of impacts (NZ$M)			Present Value of treatment costs (NZ$M)			Present value of impacts and control costs (NZ$M)		
	5%	Median	95%	5%	Median	95%	5%	Median	95%
No control	1550	2098	2560	–	–	–	1550	2098	2560
Little control	884	1441	1920	132	215	287	1016	1657	2207
Good control	27	58	113	4	9	17	31	67	129

Values are discounted at 7% and accumulated over the 30 year period. 5%, median and 95% are results for different quantiles in the stochastic spread simulations.

**Figure 8:**
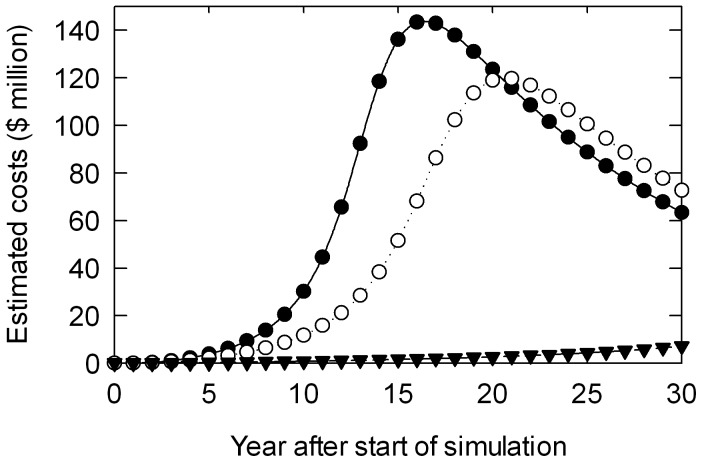
Variation in simulated annual costs due to *Thaumetopoea pityocampa* (discounted back to simulation start) under scenarios assuming no control (closed circles) little control (open circles) and good control (closed triangles). All scenarios used simulations with a median spread rate as sampled from the 50^th^ percentile.

The effect of the discounting rate interacting with the spreading impacts is apparent in the declining costs in later years in the No Control and Little Control scenarios in Figure 8. As potential impacts accrue as the pest spreads, the marginal value of these additional costs declines through time. The extreme high value in suppressing effectively the rate of spread of the pest is clearly apparent in the cost savings from effective control of the spread of *T. pityocampa* compared with doing nothing (Fig. 8).

## Discussion

### What are the Likely Costs of Invasion?

CLIMEX projections indicate that about 60% of New Zealand’s plantation forest area is climatically suitable for *T. pityocampa* under the current climate. Should this pine defoliator become established in New Zealand, outbreaks are likely to occur at a high frequency because *P. radiata* is a preferred host and it is highly susceptible to attack. This situation is likely to be exacerbated as *T. pityocampa* would experience release from the effects of at least some of its natural enemies, which contrasts with the situation in its native range [68]. If left untreated, the potential damage resulting from an invasion by *T. pityocampa* is expected to be significant (NZ$1,550 M –2,560 M, Table 3). It would appear cost-effective to spray Btk to reduce the impacts of *T. pityocampa* even with little control.

The cost estimates above only account for direct losses of stumpage value due to defoliation, and control costs to mitigate these losses. However, other damage would be incurred if this insect became established. For example, there would be considerably less carbon sequestration and loss of recreational value as well as human health costs because the urticating hairs of *T. pityocampa* cause serious dermatitis. An attempt to value these costs has been made previously [67].

Assuming that *T. pityocampa* invaded New Zealand and had become fully dispersed throughout its potential range, it would cost approximately NZ$72 M yr^−1^ to spray Btk over the 1.15 million ha of vulnerable forests. Using a 7% discount rate, these recurrent costs total NZ$966 M in PV across the thirty years of this case study. Even under the most optimistic (low cost) scenarios with a low rate of spread due to effective control (Table 3, Fig. 8), some effort at preventing the introduction and ensuring that cost-effective and socio-politically acceptable control techniques are available for deployment at short notice are clearly warranted. The amount of effort justified will also depend upon the probability of entry and establishment in New Zealand by *T. pityocampa*, [18], and consideration of the benefits of preventative measures in excluding other pests such as gypsy moth.

Accidental shipments of *T. pityocampa* egg masses with sea containers originating from infested regions are probably the highest risk introduction pathway. Larvae are vulnerable to starvation and environmental mortality factors, adults are too short-lived, and rigid biosecurity measures prevent the movement of soil that could harbour pupae. There are frequent outbreaks of *T. pityocampa* in southern Europe, which suggests that there is a high propagule pressure from its native region. These outbreaks often affect pines in urban and semi-urban areas in close proximity to industrial sites with shipping container activity [28]. It is conceivable that pine needles carrying egg masses could accidentally be transported on the surface or inside a shipping container. Incursions of several other Lepidoptera, previously considered low risk have recently occurred in New Zealand, including the white-spotted tussock moth, painted apple moth (*Teia anartoides*) and gum leaf skeletoniser (*Uraba lugens*). Even if *T. pityocampa* did not become established in New Zealand in the near future, the rate of defoliator introductions in New Zealand and overseas indicates that there is a high risk of such an invasion. Ultimately, this study should serve as a case study of a potentially invasive insect pest of pines.

The niche model indicates that the global potential distribution of *T. pityocampa* is extensive (Fig. 5), and includes most of the regions of the world where *P. radiata* is grown commercially. Interestingly, the apparently most climatically suitable areas of the world for *T. pityocampa* occur in regions outside of its native range (mostly in the southern hemisphere). These mostly sub-tropical regions experience climates that have less extreme seasonality than that of the Mediterranean basin. This curiosity may be explained by the fact that the native range of *T. pityocampa* is geographically isolated from areas with a sub-tropical climate, and should it invade such a region with a less extreme climate than its native range, it may indeed thrive. However, whilst suitable hosts are grown in these regions (e.g. *P. radiata* in eastern Australia), indicating some degree of likely climate suitability, it is prudent to treat climate suitability projections in these areas with some added caution as there may be some negative life-history factor associated with warm-wet conditions that have not been taken into account in the niche model.

### How Effective was the Modelling Method, and what did it tell us that couldn’t otherwise be Answered?

Compared with traditional economic pest risk assessments, the method demonstrated here offers significant enhancements. Traditional cost-benefit approaches ignore variability in pest population dynamics due to differences in climate throughout the area in which the vulnerable crops are grown. Typically, these analyses do not even use a species niche model for the pest to estimate which cropping regions are vulnerable to pest impacts. The CLIMEX model allowed us to identify which forests were vulnerable to attack from *T. pityocampa*, and to construct the climate-based growth impact function. Combining the 300 Index values, the CLIMEX niche model and the growth impact function allowed us to quantify potential tree growth rate impacts in a spatially-explicit and defensible manner, and to identify regions of highest priority for protection. The growth impact function is a relatively simple technique that captures climatic variability in pest suitability without resorting to the complicated and expensive method of modelling the pest population dynamics directly. By including consideration of the rate of spread of the pest, the potential costs were discounted appropriately, and the different control strategies that affect the spread rate could be evaluated.

### How Transferrable and Useful is the Method?

The data requirements for this project were significant, and it is unlikely that this type of analysis would be undertaken routinely for pest risk assessments. Of the different information components required to undertake this analysis, the CLIMEX analysis could be considered the most practiced and available. Whilst high quality fine resolution modelled productivity surfaces such as the 300 Index dataset are likely to remain rare, crop productivity maps based on crop production statistics are becoming more commonplace [e.g., www.mapspam.info (global), Eurostat (regional), and Cropscape (USA)]. The spatial resolution of the crop productivity maps are typically coarser than the 300 Index surface, though they appear adequate for combining with niche models for the type of analysis described here.

The climate-impact function is an attractive compromise between expensive detailed process-based population dynamics modelling on the one hand, and using a simplistic estimate of average pest impacts on the other. For relatively little effort, it provides a means of spatially matching pest impacts with productivity. As with the niche model, having generated the model once, it is able to be applied to pest risk assessments in any jurisdiction. However, for this type of analysis to become more commonplace, reporting of pest impact would be best studied and reported using standardised methods and measures.

Discounting the costs throughout the simulated invasion had a significant impact on the cost estimates, so it is clearly desirable to include plausible estimates of the rate of spread of invaders throughout pest risk areas. To do this routinely, the potential rates of spread of pests across landscapes may need to be better studied and documented. Whilst it is inconceivable that there would ever be a useful library of documented pest spread rates for every pest of economic concern, it is possible that a study of spread rates for different organisms could be used to develop a useful trait-based means of estimating useful rates of spread classes for invasive species.

The method of linking climate suitability, spread rates and host-impact demonstrated here appears useful and practical for estimating the potential costs of invasive pests. Whilst it has been applied to a case where impacts are considered for a single host crop, it can be easily extended to accommodate multiple hosts, so long as the impact functions can be calibrated for each crop, and the abundance of each crop is known throughout the pest risk area. The method described here can also be readily applied to pests threatening non-productive assets such as natural ecosystems using appropriate pricing mechanisms such as the hedonic pricing technique or the valuation of ecosystem services. Linking the niche modelling and economic analytical tools together as we have done in this paper provides an open and transparent means by which different pest threats can be gauged in terms of their likely economic impacts, and allows different pest threats to be compared.
